# The influence of childhood IQ and education on social mobility in the Newcastle Thousand Families birth cohort

**DOI:** 10.1186/1471-2458-11-895

**Published:** 2011-11-25

**Authors:** Lynne F Forrest, Susan Hodgson, Louise Parker, Mark S Pearce

**Affiliations:** 1 Institute of Health and Society, Newcastle University, Baddiley Clarke Building, Richardson Road, Newcastle upon Tyne, NE2 4AX, UK; 2 Departments of Medicine and Paediatrics, Dalhousie University, Halifax, Nova Scotia, Canada; 3 Institute of Health and Society, Newcastle University, Sir James Spence Institute, Royal Victoria Infirmary, Newcastle upon Tyne, NE1 4LP, UK

**Keywords:** social mobility, education, childhood IQ, social class

## Abstract

**Background:**

It has been suggested that social, educational, cultural and physical factors in childhood and early adulthood may influence the chances and direction of social mobility, the movement of an individual between social classes over his/her life-course. This study examined the association of such factors with intra-generational and inter-generational social mobility within the Newcastle Thousand Families 1947 birth cohort.

**Methods:**

Multivariable logistic regression was used to examine the potential association of sex, housing conditions at age 5 years, childhood IQ, achieved education level, adult height and adverse events in early childhood with upward and downward social mobility.

**Results:**

Childhood IQ and achieved education level were significantly and independently associated with upward mobility between the ages of 5 and 49-51 years. Only education was significantly associated (positively) with upward social mobility between 5 and 25 years, and only childhood IQ (again positively) with upward social mobility between 25 and 49-51 years. Childhood IQ was significantly negatively associated with downward social mobility. Adult height, childhood housing conditions, adverse events in childhood and sex were not significant determinants of upward or downward social mobility in this cohort.

**Conclusions:**

As upward social mobility has been associated with better health as well as more general benefits to society, supportive measures to improve childhood circumstances that could result in increased IQ and educational attainment may have long-term population health and wellbeing benefits.

## Background

Social mobility, the movement of an individual between social classes over his/her life course, is an important sociological concept in health research but also has implications for societal cohesion, equity, economic stability and happiness [1]. Socio-economic differentials in health have been identified for a wide range of health outcomes. Although the widest health gradient is generally seen between the 'socially static' [2] at either end of the social scale, the socially mobile tend to show levels of health at an intermediate level compared to their original and destination social class, so that those who move upwards are healthier than those they leave behind, but not as healthy as the group they join in most [2-4], but not all studies [5]. Mortality rates have also been shown to differ by social mobility trajectory in a similar way to morbidity [6,7]. However, it has been debated whether increasing social mobility reduces [8] or widens [2] health inequalities.

If upward social mobility can improve health and general wellbeing then this could have important policy implications, with resources targeted to help people move up the social scale or to design health promotion strategies tailored towards those groups which are immobile at the bottom. However, in order to target resources there is a need to determine the factors which are most important in facilitating mobility and also those factors that may act as barriers to mobility.

It has been suggested that social, educational, material, cultural and physical factors in childhood and early adulthood may influence the chances and direction of social mobility [9]. Education has been implicated as a predictor of upward social mobility in men in a number of studies [9-11]. Other factors postulated to influence, or act as barriers to, social mobility include childhood cognition [12], achieved adult height [3,9,12], number of siblings (as a measure of material circumstances in the family) [9,12] and changes in economic conditions [13]. Inconsistent findings in the previous studies suggest that further research on social mobility in longitudinal birth-cohorts, where study members experience economic changes at the same time, is needed. This study investigated factors in early life, childhood and early adulthood that may be drivers of, and barriers to, intra-generational and inter-generational social mobility within the Newcastle Thousand Families 1947 birth cohort.

## Methods

The Newcastle Thousand Families Study began as a prospective longitudinal study of 1142 children, born to mothers resident in the city of Newcastle upon Tyne, in northern England, in May and June 1947 [14]. The health, growth and development of the cohort were followed in great detail up to age 15 years. All families were visited both on a routine (up to every six weeks during infancy and at least quarterly until age five years) and on an *ad hoc *basis by the study team, which consisted of health visitors (nurses who visited families at home) and paediatricians. The cohort underwent a major follow-up at age 49-51 years [14]. Participants at that time were members of the cohort who were either traced through the National Health Service Central Register or contacted the study team in response to media publicity. Between October 1996 and December 1998, health and lifestyle questionnaires were sent out for completion and return and participants invited to attend a clinical assessment [14].

The study received ethical approval from the appropriate Local Research Ethics Committees and all study members gave their written consent.

### Measurement of early life experience

Information on early life was recorded prospectively for all study members [14]. Social class at age 5 years was measured using the Registrar General's definition of occupational social class of head of household, with social class split into six categories ranging from I (professionals, assumed to be the most advantaged), II (managerial and technical), IIIn (skilled non-manual), IIIm (skilled manual), IV (partly skilled manual) to V (unskilled manual, assumed to be the least advantaged).

Housing conditions were recorded by the City Public Health Department in 1952 based on four measures of living standards that assessed whether participants lived in households that were: a) overcrowded; b) lacked hot water; c) shared a toilet or d) where the house was damp or in poor repair. A composite housing condition variable recorded the number of adverse housing conditions suffered, from 0 to 4. As low numbers were found in some categories this variable was dichotomised to 'no housing problems' (score 0) or 'some housing problems' (score 1-4).

Adverse events suffered in the first five years of life was a composite variable examining deficiency of care and social dependence using five measures of family life standards consisting of: a) serious parental debt; b) parental divorce or separation; c) parental incapacity through chronic illness; d) parental criminal activity or cruelty; e) death of a parent. Again as low numbers were found in some categories this variable was dichotomised to 'no adverse events' (score 0) or 'some adverse events' (score 1-5).

In 1958, study members took the 11-plus examination, consisting of written papers involving tests of verbal reasoning (Moray House tests 57 and 58) and two standardized tests of English and arithmetical ability [15,16]. The total IQ score was derived as the average of the four test results. At that time in England, the 11-plus examination was a standard test used in educational establishments at the age of 11 years, often to determine the type of secondary school at which a child was to continue their education.

### Measurement of adult socioeconomic position, social mobility and other adult data

Social class was prospectively derived at age 49-51 years and retrospectively derived for age 25 years from the health and lifestyle questionnaire, using occupational details of the main wage earner in the household coded according to the 1990 UK Registrar General's Standard Occupational Classification, again with I assumed to be the most advantaged and V the least advantaged. Due to the small numbers in some occupational social classes, social class was collapsed into four groups-Group 1 consisted of occupational social classes I and II, group 2 of social class III (non-manual), group 3 of social class III (manual) and group 4 of social classes IV and V.

Inter-generational social mobility (between parental social class and individuals' own social class in adulthood) was measured between ages 5 and 25 years and between 5 and 49-51 years, and intra-generational mobility between ages 25 and 49-51 years. Within these three timescales, four potential socio-economic trajectories categories were created: a) 'stable manual': those remaining in social class group 3 or remaining in social class group 4; b) 'stable non-manual': those remaining in social class group 1 or remaining in social class group 2; c) 'upward mobility': any move upward from a lower to higher social class group and d) 'downward mobility': any move downward from a higher to lower social class group.

Highest achieved education level, ascertained from the health and lifestyle questionnaire at age 40-51 years, was grouped as follows: a) no formal qualifications; b) O levels/apprenticeship/clerical qualifications; c) A levels/HNC/other similar level qualification and d) degree or postgraduate qualification. Achieved adult height in centimetres was as measured at the clinical assessment at age 49-51 years [14].

### Statistical analysis

Representativeness of the participants in this study compared to those of the original cohort not included was assessed using t, Mann Whitney and χ^2 ^tests as appropriate. Twins (n = 14) were excluded from all analyses due to their potential lack of independence in statistical models.

All analyses of socio-economic trajectory were done over three timescales (age 5 to 49-51 years, 5 to 25 years and 25 to 49-51 years). Achieved adult height and childhood IQ were treated as continuous variables, the other variables as categorical. Multivariable logistic regression was used to examine the potential associations of childhood IQ, education level, achieved adult height, housing conditions at age 5 years, adverse events in early childhood and sex with upward and downward mobility. Likelihood ratio tests were used to assess the significance of each variable. The odds of upward and downward mobility were examined, with upward and then downward mobility compared against all other possible trajectories i.e. those who were not upwardly (or downwardly) mobile. Odds ratios (OR) and corresponding 95% confidence intervals (95% CI) are reported.

For upward mobility between 5 and 25 years and between 5 and 49-51 years those who were in social class 1 at age 5 were excluded as they were unable to experience upward mobility and likewise those in class 4 at age 5 were excluded for downward mobility. Similar exclusions were applied at age 25 years for mobility between ages 25 to 49-51 years. In addition to combined analyses, upward and downward mobility were also examined separately for men and women over each timescale, as sex-specific effects of social mobility on health have been previously reported for this cohort [4,13,17]. Regression analysis was performed for all potential factors simultaneously to produce a multivariable model for each timescale, to take account of potential confounding factors. As adult height was measured at the clinical assessment and thus not available for the entire sample, models were established without height at first, and then adjustment for height tested on the smaller sample for which height was available. Potential interactions were tested within the regression modelling framework.

The statistical software package Stata (version 10.0; StatCorp, College Station, TX, USA) was used for all statistical analyses.

## Results

Table 1 shows socio-demographic characteristics of the study population at baseline (age 5 years) and at follow-up (ages 49-51 years); 789 participants had social class data at age 5, 574 participants completed questionnaires at age 49-51 years, and 457 had social mobility data from age 5 to 49-51 years (451 from 5 to 25 years, and 499 from 25 to 49-51 years). Table 2 summarises the descriptive data for each potential driver of social mobility by social mobility trajectory between 5 and 49-51 years and for the cohort overall. There was no significant difference in terms of social class at age 5 years between those included in this study and the remainder of the cohort (p = 0.45). A significantly higher proportion of women were included in this study than in the remainder of the cohort (p < 0.01). Those who remained in the study had significantly higher median childhood IQ than those who did not (p < 0.01) and those who had housing problems in childhood were under-represented (p = 0.02).

**Table 1:** Descriptive characteristics of cohort with social mobility data from ages 5 to 49-51 years

Variable	N	(%)
**Sex**		
Male	214	(46.8)
Female	243	(53.2)
Total	457	

**Social class at age 5**		
I/II	39	(8.5)
IIIn	64	(14.0)
IIIm	198	(43.3)
IV/V	156	(34.1)
Total	457	

**Social class at age 49-51**		
I/II	227	(49.7)
IIIn	54	(11.8)
IIIm	106	(23.2)
IV/V	70	(15.3)
Total	457	

**Table 2:** Descriptive summary of potential factors associated with social mobility, by social mobility trajectory from age 5 to 49-51 years

	IQ		Education level:		highest qualification			Height (cm)	Housing problems		Adverse events	
						
			None	O level	A level	University			None	Some	None	Some
**Trajectory**	**N**	**Median (IQR)**	**N (%)**	**N (%)**	**N (%)**	**N (%)**	**N**	**Mean (SD)**	**N (%)**	**N (%)**	**N (%)**	**N (%)**

**Stable non-manual**	30	113 (100-124)	4 (11.1)	12 (33.3)	8 (22.2)	12 (33.3)	30	167.00 (9.14)	30 (75)	10 (25)	25 (78.1)	7 (21.9)
**Upward mobility**	267	104 (93-113)	81 (30.7)	92 (34.9)	54 (20.4)	37 (14.0)	214	166.90 (8.79)	157 (55.7)	125 (44.3)	161 (59.9)	108 (40.1)
**Downward mobility**	41	94 (87-103)	20 (43.5)	18 (39.1)	6 (13.0)	2 (4.3)	31	165.08 (7.74)	27 (57.5)	20 (42.5)	25 (61)	16 (39)
**Stable manual**	73	93 (85-102)	47 (61.8)	22 (29.0)	6 (7.9)	1 (1.3)	57	166.45 (8.04)	30 (37.5)	50 (62.5)	36 (48)	39 (52)

**Total**	411	102 (90-112)	152 (36.0)	144 (34.1)	74 (17.5)	52 (12.3)	332	166.66 (8.59)	244 (54.3)	205 (45.7)	247 (59.2)	170 (40.8)

### Upward mobility

Childhood IQ and achieved education level were both significantly, and independently, positively associated with upward mobility from 5 to 49-51 years in the model containing all variables except height (table 3). Following further adjustment for height, education level remained significant but IQ lost significance. Whilst IQ and education level were significantly associated with upward mobility between ages 5 and 25 years at the univariable level, only education level remained significant in the multivariable model. Only childhood IQ was significantly associated with upward mobility between ages 25 and 49-51 years, in the multivariable model. Adjusting for adult height made very little difference to these results.

**Table 3:** Results of logistic regression analysis between childhood IQ and education level and upward mobility

	Uni variable analysis				Multi variable analysis *		
	**N**	**OR**	**95% CI**	**P**	**OR**	**95% CI**	**P**

**Age 5 to 49-51**							
**IQ**	383	1.05	1.04-1.07	< 0.001	1.03	1.01-1.05	0.004
**Education level:**	387			< 0.001			0.006
None		1.00			1.00		
O level		1.86	1.14-3.03		1.56	0.88-2.73	
A level		3.72	1.84-7.53		2.59	1.15-5.83	
University		10.20	3.01-34.56		8.06	1.71-37.84	

**Age 5 to 25**							
**IQ**	382	1.04	1.02-1.05	< 0.001	1.01	0.993-1.04	0.20
**Education level:**	388			< 0.001			0.005
None		1.00			1.00		
O level		1.61	0.99-2.60		1.47	0.84-2.56	
A level		6.21	2.66-14.52		5.11	1.93-13.53	
University		6.79	2.30-20.07		6.09	1.60-23.17	

**Age 25 to 49-51**							
**IQ**	261	1.05	1.03-1.07	< 0.001	1.05	1.02-1.07	< 0.001
**Education level:**	283			0.19			0.39
None		1.00			1.00		
O level		1.16	0.68-1.98		0.80	0.42-1.51	
A level		2.06	0.98-4.36		1.24	0.48-3.21	
University		2.15	0.68-6.79		2.89	0.56-14.95	

*Adjusted model including sex, housing conditions and adverse events in childhood

In men, higher childhood IQ and achieved education level were significantly associated with increased odds of upward mobility between ages 5 and 49-51 years at the univariable level (table 4). However, only IQ remained significant in a model containing both variables (OR per IQ unit 1.04, 95% CI 1.004-1.07, p = 0.03). For upward mobility between ages 5 and 25 years in men; IQ (p = 0.002), and education level (p = 0.006) were significant at the univariable level, but were not significant after co-adjustment. For upward mobility between 25 and 49-51 years, only childhood IQ was significant in a model also containing achieved education level (OR per IQ unit 1.05, 95% CI 1.02-1.09, p = 0.01).

**Table 4:** Results of univariable logistic regression analysis of upward mobility between ages 5 and 49-51 years, for men and women

			Men				Women	
	N	OR	95% CI	P	N	OR	95% CI	P
**IQ**	176	1.05	1.02-1.08	< 0.001	207	1.06	1.03-1.09	< 0.001

**Education level:**	175			0.002	199			< 0.001
None		1.00				1.00		
O level		1.43	0.66-3.09			2.39	1.24-4.59	
A level		2.95	1.20-7.27			8.60	1.92-38.60	
University		7.38	1.97-27.71			α	---	

**Housing conditions:**	187			0.22	223			0.19
No problems		1.00				1.00		
1-4 problems		0.68	0.37-1.25			0.68	0.38-1.21	

**Adverse events:**	176			0.35	212			0.28
None		1.00				1.00		
Some (1-5)		0.74	0.39-1.40			0.72	0.40-1.30	

**Height (cm)**	133	1.04	0.98-1.10	0.24	170	1.00	0.94-1.05	0.89

In women, higher childhood IQ and achieved education level were significantly associated with increased odds of upward mobility from 5 to 49-51 years at the univariable level (table 4), although only IQ remained significant in the multivariable model. As all upwardly mobile women had a university-level education this resulted in infinite odds. In women, therefore, university-level education was associated with upward mobility, but education level itself was not (p = 0.08), because the university-level women were excluded from the model due to a lack of variation in the mobility outcome data for these women. For upward mobility between ages 5 and 25 years; childhood IQ (p = 0.005), and education level (p < 0.001) were significant at the univariable level, but only education level was significant (p < 0.001) in the multivariable analysis. This remained the case when those with university- level education were excluded from the analysis. For upward mobility between ages 25 and 49-51 years, only childhood IQ was significant (OR per IQ unit 1.03, 95% CI 1.00-1.07, p = 0.05), in a model with education, although again all upwardly mobile women had university-level education.

### Downward mobility

Whilst childhood IQ and education level were significant predictors of downward mobility between ages 5 and 49-51 years at the univariable level (table 5), only low childhood IQ retained significance in the multivariable model (OR 0.95 per IQ unit 0.95, 95% CI 0.92-0.99, p = 0.006). When examined by sex, only childhood IQ was significantly associated with downward mobility for men (OR per IQ unit 0.90, 95% CI 0.85-0.96, p = 0.001). For women childhood IQ was significant at the univariable level (OR per IQ unit 0.96, 95% CI 0.93-0.995, p = 0.03), but lost significance in the adjusted models. No women with a university education were downwardly mobile and so were this group was excluded from the analysis due to a lack of variation in outcome data.

**Table 5:** Results of univariable logistic regression analysis of downward mobility between ages 5 and 49-51 years

	N	OR	95% CI	P
**IQ**	265	0.95	0.92-0.97	< 0.001

**Education level:**	275			0.009
None		1.00		
O level		0.67	0.33-1.38	
A level		0.35	0.13-0.95	
University		0.14	0.03-0.65	

**Housing conditions:**	294			0.68
No problems		1.00		
1-4 problems		1.15	0.61-2.16	

**Adverse events:**	268			0.72
None		1.00		
Some (1-5)		1.13	0.57-2.24	

**Height (cm)**	219	0.97	0.93-1.01	0.18

**Sex:**	294			0.12
Male		1.00		
Female		1.67	0.87-3.18	

Lower childhood IQ was significantly associated with increased odds of downward mobility between 5 and 25 years in the unadjusted analysis (OR per IQ unit 0.96, 95% CI 0.94-0.99, p = 0.013) but lost significance in the adjusted analysis. The same pattern was seen when restricting the analysis to just women, but for men, no significant associations were seen. Lower childhood IQ and lack of higher-level qualifications were significantly associated with increased odds of downward mobility between ages 25 and 49-51 years at the univariable level. However, only IQ remained significant in the adjusted model (OR per IQ unit 0.97, 95% CI 0.94-0.99, p = 0.002). Low childhood IQ was significantly associated at the univariable level with an increased odds of downward mobility for both men and women, but remained significant for only the men in the adjusted analyses (OR per IQ unit 0.96, CI 0.93-0.996, p = 0.03).

## Discussion

In this cohort, childhood IQ and achieved education level were both positively associated with increased odds of upward mobility between the ages of 5 and 49-51 years. However, only education level was significantly associated with upward mobility between 5 and 25 years and only childhood IQ was significantly associated with upward mobility between 25 and 49-51 years. Lower childhood IQ was associated with increased odds of downward mobility.

As previously noted in studies of social mobility and health in this cohort [4,13,17], most mobility was in an upward direction. This reflects changes in occupational social class patterns found throughout the UK [9], where there was a considerable amount of absolute intergenerational social mobility over this period. Economic and social change meant that there was 'more room at the top' [1]. For the Newcastle Thousand Families birth cohort, all born in 1947 in a city in the north of England [14], their career and educational opportunities are likely to have been far greater than that experienced by their parents as, although manufacturing has declined, there has been a massive growth in the service sector leading to a much larger middle class and the size of the unskilled manual labour force has decreased.

In this study, higher achieved education level was a significant driver of upward mobility. Education has been described as 'the main engine of social mobility' [18] and as a means to 'escape from childhood disadvantage' [19]. Education has been associated with social mobility in men in a number of previous studies [9,10,12], measured either as number of years of education or as level of education, but only one of these studies also measured IQ. In that study high IQ was associated with upward mobility at the univariable level, but the association was lost in the multivariable model when education, height and number of siblings were included [12]. However, men who were in an advantaged social class in adulthood had the highest childhood IQ scores, irrespective of their fathers' social class, suggesting that IQ may be important for upward social mobility [12]. In the current study, whilst higher childhood IQ and education level were significant predictors of upward mobility between 5 and 49-51 years at the univariable level in men, only IQ was significant in the adjusted model, although this may have been due to the low numbers of men investigated.

High childhood IQ was significantly associated with upward mobility between ages 5 and 49-51 years and between 25 and 49-51 years in this cohort. Our findings in relation to childhood IQ and social mobility support previous evidence suggesting that IQ level is more strongly associated with social class attained in middle-age rather than early adulthood and that people with high IQs eventually move up the occupational ladder regardless of their educational qualifications [12]. However, education does appear to be important for early mobility as only education level was significant for early social mobility between the ages of 5 and 25 years. As in previous studies [11], IQ and educational achievement were correlated. Higher childhood IQ has been associated with higher age of leaving education for men [12] who are therefore likely to obtain higher level qualifications, but this may also depend on the social circumstances of the family.

In a meritocracy, individuals should be able to improve their circumstances through ability and effort. There is a split in the literature between those who believe that the UK is 'to a large extent a meritocratic society' [20] where 'IQ plus effort' can allow someone to advance up the social scale, and those who argue that children from more disadvantaged origins 'need to show substantially more merit' [21] in order to reach the same levels. Results from this study suggest that higher IQ in childhood is associated with upward mobility, supporting the meritocracy theory that 'IQ plus effort' does indeed allow upward mobility. However, in the descriptive analyses the highest IQ levels were found in the stable non-manual group rather than the upwardly mobile, a pattern that has been reported elsewhere [22]. Therefore, it appears that those from more disadvantaged backgrounds in this study do not need to exhibit 'more merit' than those who originate from a higher social class, in order to reach the same broad level on the social scale. However, we cannot say anything about finer stratification within each occupational social class, where evidence still suggests that those from more privileged backgrounds tend to occupy the more lucrative and prestigious professions [23].

Low childhood IQ [12] and low achieved education levels [10,12] have previously been associated with downward mobility in men, although only one study considered both [12]. In the current study, those with a lower childhood IQ were significantly less likely to be upwardly mobile and more likely to be downwardly mobile, suggesting that low childhood IQ is both a barrier to upward mobility and a driver of downward mobility. Education level was not significantly associated with downward mobility.

Previous studies have largely focused on men, and social mobility in women has not been explored. In this study childhood IQ was significantly associated with upward mobility between 5 and 49-51 years and between 25 and 49-51 years for women. University education also appeared to be important for women in achieving upward social mobility as all women in this cohort who had a university-level education were upwardly mobile. Education level, but not childhood IQ, was significant for upward mobility in women between ages 5 and 25 years.

It was found that, for each level of education, women had a higher median IQ than men. Therefore it appears that women in this study, who were as intelligent as men, did not achieve the same level of education. Members of this cohort were teenagers in the 1960s when only a small percentage of the population went to university and there were still high levels of inequality between the sexes. It is possible that the observed sex differences are due to the social values of the time, where education was not considered as important for women. However, for those women who did go on to higher education this allowed them to progress up the social scale or maintain their high social position, as university degree-level or post-graduate qualifications were only found in stable non-manual or upwardly mobile women.

It has been suggested that educational success might not necessarily result in upward mobility if many disadvantages have been suffered in childhood [19]. However, very few data are available on the influence of childhood social circumstances on social mobility. In this current study where it was possible to examine this, adverse circumstances (including parental debt, death, divorce and imprisonment) and poor housing conditions in childhood did not significantly affect the chances of social mobility in either direction.

In children who experience deprivation, those who are most advantaged in terms of social capital are more likely to be upwardly mobile [24]. Social capital can be measured in material, psychological and cultural terms [25]. Conditions in childhood which result in low levels of social capital might also contribute to shorter stature [25] so that height can be used as a proxy measure of childhood circumstances as inadequate nutrition contributes to attained adult height [12]. Achieved adult height has been directly linked to social mobility in men [3] with taller men who were born between 1905 and 1935 [9] and who were born in 1921 [12] showing upward mobility, with downward mobility seen for those who were shorter. In the current study, with participants born in 1947, height was not significantly associated with social mobility. There is a lack of published data relating to height and social mobility in women. Here, no association was found between height and social mobility in women. It is possible that due to the post-war conditions that the cohort grew up in, with rationing still in place until 1954, potential height differences by social class were reduced, resulting in non-significant differences in height between socioeconomic trajectories, as found here for both men and women.

The main strength of this study is the prospective nature of the data and the extensive follow up covering 50 years, which allows analysis of both inter-generational and intra-generational social mobility. The study examined social mobility in women as well as men, something that has not been done before, although numbers were low when the sexes were examined separately. Some retrospective data were obtained to determine social class at age 25 years, but all childhood and age 50 data were prospectively collected thus reducing the possibility of recall bias. The wide ranging available data meant that the indirect selective effects of social, biological and educational factors on social mobility could all be examined simultaneously. However, whilst the range of data examined allowed confounding to be taken into account, the possibility of residual confounding having a role in interpreting the findings remains.

As data on social factors such as adverse circumstances and housing conditions in childhood were available, it was possible to examine whether those who lived in more deprived circumstances achieved their educational potential and whether this influenced social mobility. IQ data were collected prior to educational attainment and the influence of both was evaluated, which allowed consideration of whether those from lower social class backgrounds need to show 'more merit' than those from more privileged households in order to obtain similar adult socio-economic standing. As the cohort members were aged 49-51 years at the time of data collection for this study, it was possible to collect details of educational attainment achieved later in life. Social class at age 5 years rather than at birth was used. With the UK recovering from war in 1947, many of the occupations at the time may not have truly represented the head of household's true standing. Taking social at age 5 years is likely to give a more accurate picture of the individual's childhood socio-economic circumstances than using birth data.

Loss to follow-up can be an issue in longitudinal cohort studies and is not always randomly distributed. Here, there was no significant difference in childhood social class between those included in this study and the remainder of the cohort. A significantly higher proportion of women were included in this study than in the remainder of the cohort, but the sexes were examined separately.

The problems inherent in assigning a SES based on occupation to women should also be acknowledged, particularly when using the highest occupational social class in the household which, for many women in this cohort, resulted in them being assigned the SES of their male partner. However, the use of husband's occupation to determine social class for women can also be regarded as a strength, as it is debateable how relevant a women's own occupation actually is as a reflection of socio-economic status, as this tends to be compromised by motherhood [8]. Becoming a mother can often result in apparent downward social mobility for women [10] as they move from an employed to a caring role. We did not have data on the number of women in this cohort for whom this was the case, but it is likely that many of these women did not work or gave up work when they had children [17]. Therefore the husband/partner's SES is more likely to truly reflect the SES of the household.

Those who remained in the study had significantly higher median childhood IQ than those who did not. Low childhood IQ in this cohort has been associated with an increased risk of early mortality [16], which might partially explain why those who remained had higher IQ. Low IQ has also previously been associated with increased risk of morbidity [26] and with adverse health behaviours [26].

Although this study was able to include some adverse social and material circumstances in childhood, the fact that these markers of deprivation and adversity were not associated with social mobility in this cohort might suggest that other measures of social support that we were not able to determine may play a role in limiting some of the adverse effects of socio-economic disadvantage in childhood. This could be an area for future research in this cohort.

## Conclusions

This study has shown associations between both childhood IQ and achieved education level and the odds of upward and downward mobility. Material factors such as poor household living conditions and adverse psychological experiences in childhood (such as the death or imprisonment of a parent) were not associated with mobility in this cohort and those who experienced childhood adversity were still able to move up the social scale due to aptitude and ability.

The factors that influence IQ and educational attainment may be the type of social capital measures that are also likely to result in upward mobility, although there is some debate as to whether IQ level is alterable [27,28]. IQ may have some genetic heritability, but the environment also appears to play a role [28] and factors such as parental child-rearing style and the number of books in the home have been shown to influence IQ in young children [29]. Likewise, the value placed on education, the availability of books in the home and parental support and encouragement to stay on at school have all been suggested as factors that might determine whether someone is able to achieve their educational potential [19]. IQ and educational attainment are likely to influence employment prospects and thus adult SES [16].

Upward social mobility has been associated with better health as well as more general benefits to society in terms of population happiness, equity and equality of opportunity [1,18]. As both high IQ and education level are associated with upward mobility, supportive measures to improve childhood circumstances that could result in increased IQ and educational attainment may have long-term population health and wellbeing benefits.

## List of abbreviations

CI: Confidence interval; IQ: Intelligence quotient; OR: Odds ratio.

## Declaration of competing interests

The authors declare that they have no competing interests.

## Authors' contributions

MSP and SH conceived the study and supervised LFF who analysed the data under their supervision as part of her Masters project. LP was responsible for the data collection during the age 49-51 years follow-up. LFF wrote the first draft of the manuscript with assistance from MSP and SH. All authors commented critically on drafts of the manuscript and all read and approved the final manuscript.

## Pre-publication history

The pre-publication history for this paper can be accessed here:

http://www.biomedcentral.com/1471-2458/11/895/prepub
